# Retraction: Transient activation of the PI3K/Akt pathway promotes Newcastle disease virus replication and enhances anti-apoptotic signaling responses

**DOI:** 10.18632/oncotarget.28683

**Published:** 2025-01-20

**Authors:** Yinfeng Kang, Runyu Yuan, Xiaqiong Zhao, Bin Xiang, Shimin Gao, Pei Gao, Xu Dai, Minsha Feng, Yanling Li, Peng Xie, Yulian Li, Xiaoyi Gao, Tao Ren

**Affiliations:** ^1^Key Laboratory of Zoonosis Prevention and Control of Guangdong Province, College of Veterinary Medicine, South China Agricultural University, Guangzhou, 510642, China; ^2^State Key Laboratory of Oncology in South China, Collaborative Innovation Center for Cancer Medicine, Department of Experimental Research, Sun Yat-sen University Cancer Center, Guangzhou, 510060, China; ^3^Key Laboratory of Animal Vaccine Development, Ministry of Agriculture, Guangzhou, 510642, China; ^4^Key Laboratory for Repository and Application of Pathogenic Microbiology, Research Center for Pathogens Detection Technology of Emerging Infectious Diseases, Guangdong Provincial Center for Disease Control and Prevention, Guangzhou, 510300, China; ^5^College of Animal Science and Technology, Shanxi Agricultural University, Jinzhong, 030800, China

**This article has been retracted:** Oncotarget has completed its investigation of this paper. This investigation was initiated at the request of the corresponding author, who stated: “we performed a review of the supporting data and research records and determined that the research was not performed according to expected standards and was not reliable.” The investigation found that several images within the paper have been manipulated and duplicated. Specifically, Figure 1, panel B representing western blot Akt bands was duplicated in Figure 10C as GAPDH bands. The Akt panel in Figure 2A is also a duplicate of the Akt panel in Figure 2C, which represents a different experiment. Additionally, the pAkt panel in Figure 3A was manipulated and presented as the Akt western blot panel of Figure 7A. The authors also reused some western blot and Flow cytometry images to illustrate different experiments in the later published paper [1]. In light of these facts, the Editorial decision was made to retract this paper. The authors were contacted regarding this retraction, and a request for signatures authorizing the retraction was made, but no signatures were received.

Original article: Oncotarget. 2017; 8:23551–23563. 23551-23563. https://doi.org/10.18632/oncotarget.15796
